# Precarious employment and mental health in the Belgian service voucher system: the role of working conditions and perceived financial strain

**DOI:** 10.1007/s00420-024-02057-z

**Published:** 2024-03-26

**Authors:** Christophe Vanroelen, Eva Padrosa Sayeras, Jessie Gevaert, Kelly Huegaerts, Mattias Vos, Kim Bosmans

**Affiliations:** 1 Brussels Institute for Social and Population Studies, Vrije Universiteit Brussel, Brussels, Belgium; 2 ESIMar (Mar Nursing School), Parc de Salut Mar, Universitat Pompeu Fabra-Affiliated, Barcelona, Spain; 3 SDHEd (Social Determinants and Health Education Research Group), IMIM (Hospital del Mar Medical Research Institute), Barcelona, Spain; 4 GREDS-EMCONET (Research Group On Health Inequalities, Environment, Employment Conditions Network), Universitat Pompeu Fabra, Barcelona, Spain

**Keywords:** Precarious employment, EPRES, Mental health, Domestic cleaners, Belgium, Job quality

## Abstract

**Introduction:**

Jobs in domestic cleaning are often conceived as ‘precarious employment’ (PE)—i.e. a multidimensional concept referring to accumulated adverse characteristics of employment due to workers’ weak bargaining position. Against this background, the Belgian service voucher system (SVS) was implemented aimed at creating formal and stable, subsidized domestic services jobs.

**Purpose:**

The current study assesses the relationship between PE and mental health (WHO5) in the Belgian SVS, accounting for the potential mediating role of working conditions and perceived financial strain at the household level.

**Methods:**

We analysed a cross-sectional sample of 1,115 Belgian SVS domestic cleaners, collected in 2019 through an online survey. A mediation model was estimated.

**Results:**

The crude effect of PE on adverse mental health was strong (ß 0.545—S.E. 0.063). However, 50% of the association between PE and mental well-being was mediated by work task characteristics (quantitative demands, physical demands, task variation and autonomy) and 25% by household-level perceived financial strain. The remaining direct effect of PE on adverse mental well-being is ß 0.066 (S.E. 0.032—25% of the total effect).

**Conclusion:**

These findings are the first based on the Belgian Employment Precariousness Scale (EPRES-BE) and are consistent with earlier-made—but seldom simultaneously tested—assumptions on the mechanisms relating PE to adverse mental health—i.e. involving direct associations and indirect associations via adverse working conditions and material deprivation. Based on the results, we recommend more democratic and higher-quality management practices in the SVS, in addition to higher wages and working time reduction.

**Supplementary Information:**

The online version contains supplementary material available at 10.1007/s00420-024-02057-z.

## Introduction

The mid-twentieth century gendered division of labour traditionally entrusted men with the bulk of productive activities, while unpaid domestic work was attributed to women. With the feminization of the labour market and the increase in dual-earner families since the last quarter of the twentieth century, service structures emerged that bring domestic tasks in middle-class families to the paid labour market. Women, often socially disadvantaged according to several axes of social inequality (e.g. social class, migrant background, single parentship), constitute the core of the domestic workforce that emerged (Giordano 2019). Because domestic work was historically non-professionalized, it is popularly perceived as easy work with low skill requirements which are almost innate in women (Bosmans et al. 2016). This leads to a high likelihood for workers in the domestic sector to find themselves in precarious employment, defined as an accumulation of unfavourable employment conditions and relations (Jokela 2019). Domestic work is known for its high concentration of undeclared work (European Union 2020), which implies a lack of social protection and basic workers’ rights, as well as dependent, servile employment relationships (Bosmans et al. 2016). Domestic cleaners not only face adverse employment conditions and relations, but also particularly taxing work task-intrinsic characteristics: high physical and psychological demands, toxic exposures, dangerous situations, repetitive movements, awkward bodily positions, and social isolation (Ish et al. 2020; Malhotra et al. 2013). This leads to a high prevalence of various work-related health conditions, including respiratory problems, back pain and other musculoskeletal complaints, occupational accidents, and cancers (Archangelidi et al. 2021; Dumas et al. 2013; Jebaraj et al. 2022; Malhotra et al. 2013). Also mental health complaints are a concern among domestic cleaners (Ish et al. 2020). Likely drivers of mental health problems among domestic workers are adverse (psychosocial) working conditions and structural factors like material deprivation (Guerra et al. 2022).

Against this unfavourable background, a major policy aim of several EU-member states regarding the domestic sector consists of the transformation of undeclared work into the formal economy and by that granting minimum wages, social protection, basic OSH-compliance and a general improvement of the working conditions (Kvist 2012). ‘Service voucher schemes’—a means of payment subsidized by government—are one of the possible incentives to realize this policy aim at the ‘demand side’ of the labour market (Williams 2018). The Belgian service voucher system (SVS), implemented in 2004, figures as an example in case for this practice that has also been implemented in other EU countries, including France, Germany, Finland and Austria (Kvist 2012; Williams 2018). The Belgian SVS is a mixed public–private employment scheme for predominantly (but not exclusively) domestic cleaning services, where service providers (SVS agencies) manage and dispatch workers to clients (households). The Belgian SVS serves a threefold policy aim: to formalize paid domestic work, to generate employment opportunities and easy labour market access for low-skilled workers, and to provide feasible solutions to the work–life conflict of middle-class dual breadwinner families (Goffin et al. 2018). Because of the very cheap prices for households (a net cost of between 6 and 7.2 euros), the SVS became a very successful employment scheme. In 2021, it employed around 150,000 full-time or part-time workers, serving more than 20% of all Belgian households. The contracted services are mainly domestic cleaning, although also other services are offered (e.g. ironing, doing groceries, or preparing meals). Belgian authorities heavily subsidize the SVS (ca. 2 billion euros yearly), leading to discussions on the effectivity and efficiency of the scheme (Adriaenssens et al. 2023; Lens et al. 2022).

By bringing domestic cleaning to a formal employment setting, the SVS aimed to reduce employment precarity and consequently improve general working conditions (Mousaid et al. 2017). Evaluation studies have shown that SVS employment is certain, stable, secure and grants domestic cleaners access to the social benefits and statutory entitlements of regular wage earners (Lens et al. 2021; Quintelier 2020). This is quite an achievement when compared with the employment situation of domestic cleaners globally. Nevertheless, SVS employment remains being rather low-quality work. It is important to consider the mere nature of the domestic work tasks (e.g. physical and psychological task demands), the low status and ‘servile’ employment relationships that constitute a likely less healthy (psychosocial) work environment, compared to other job settings (Mousaid et al. 2017; Safuta & Camargo 2019). Moreover, SVS workers get a gross hourly wage of around 13 euros, which is well below the median gross hourly wage in Belgium (around 23 euros) and only a bit higher than the national minimum wage. Many SVS workers still face income insecurity because of the high prevalence of part-time work and regular fluctuations in their monthly income due to cancelled or additional cleaning jobs (Mousaid et al. 2017). Other findings refer to the huge power inequality between SVS workers and their employing agency, lack of collective voice, lack of compliance with regulations, low investment in training, and conflicts with the employing agencies and clients (Michielsen 2018; Mousaid et al. 2017). Previous research has also shown that employment conditions may vary between SVS workers, which likely reflects differences in the management quality across different SVS employment offices and in the quality of the relationships with clients (Mousaid et al. 2017; Safuta and Camargo 2019).

The large number of workers involved, the evident OSH risks associated with domestic cleaning, combined with the highly female, low-skilled and migrant workforce, makes the quality of work in the SVS a primary public health concern (Mousaid et al. 2015). This makes the SVS an interesting case for studying the relation between precarious employment and (mental) health. Therefore, the general objective of the current study is to assess the relationship between precarious employment and mental health among Belgian SVS workers, accounting for the potential mediating role of working conditions and perceived financial strain at the household level.

## Measuring precarious employment using the EPRES-BE scale

Precarious employment is an emerging concept in occupational health research given the emerging evidence for its harmful effects on workers’ health and well-being (Vanroelen et al. 2021). While it has been conceptualized in many forms (Kreshpaj et al. 2020), there is growing consensus on its multidimensional character. More specifically, precarious employment can be seen as an accumulation of unfavourable employment conditions and relations that are essentially due to workers’ weak bargaining power (Vanroelen et al. 2021). In analytical terms, this implies that precarious employment does not encompass task-intrinsic job characteristics (e.g. job content or task demands), but rather issues related to employment conditions (e.g. the stability of employment and income, access to rights and benefits, access to opportunities, or working hours arrangements) and employment relations (e.g., voice and participation in decision-making, interpersonal relations with superiors) (Rubery et al. 2018). According to Rubery et al. (2018), ‘favourable’ employment conditions and relations’ present ‘objective benefits’ for workers of the so-called standard employment relationship (SER)—i.e. the stable, full-time and socially protected model of salaried employment that came into effect in the West after the Second World War. Although domestic work constitutes a labour market niche that never got integrated into the SER-model, this model can be used as a kind of gold standard for assessing the degree of precariousness of actual employment arrangements, also in domestic cleaning.

Several ways to conceptualize and empirically assess precarious employment and its consequences exist (Kreshpaj et al. 2020). This paper adheres to the ‘EPRES-tradition’ in employment precariousness research. EPRES stands for ‘Employment Precariousness Scale’ and can be seen as an approach to conceptualize and empirically assess the degree of employment precariousness and its consequences (Vives et al. 2010). The approach gained traction in social epidemiological research (Vanroelen et al. 2021). The instrument has been applied in several European and non-European countries, including Spain (Vives et al. 2015), Greece (Maria et al. 2019), Sweden (Jonsson et al. 2019), Belgium (Vandevenne et al. 2022), Chile (Vives-Vergara et al. 2017), Finland (Hult et al. 2023) and the USA (Han and Hart 2021), as well as in a European cross-country study using secondary data (Padrosa et al. 2021).

In this paper, we will make use of the Belgian adaptation, EPRES-BE, consisting of eight dimensions: (1) temporariness (i.e. the contractual duration of the employment relation), (2) disempowerment (i.e. lack of participation and voice on wages, benefits and work planning), (3) vulnerability (i.e. being exposed to authoritarian treatment, lack of information or incorrect administration of wages), (4) rights (i.e. lack of entitlement to basic work-related rights), (5) enforceability of rights (i.e. not being able to execute the work-related rights one is entitled of), (6) working times (i.e. long, flexible and/or unpredictable working times), (7) wages (i.e. low monthly net wages) and (8) training (i.e. lack of formal training provided by the employer) (Vandevenne et al. 2022). The dimensions of ‘working times’ and ‘training’ were not included in the original EPRES. However, good arguments in favour of including both dimensions have been formulated by authors who applied the EPRES-framework to the cross-national data of the European Working Conditions Surveys: unpredictable and flexible working times and limited training opportunities can both be conceived as breaches with the SER-model of negotiated, predictable and long-term-oriented employment (Padrosa et al. 2021; Van Aerden et al. 2014). Therefore, we decided to include both additional dimensions in the EPRES-BE.

## Linking precarious employment to mental health among domestic cleaners

Precariousness employment, independent from the way it is measured, is found to be associated with adverse mental health (Jaramillo et al. 2022). Also, studies using the EPRES-scale, have generally pointed towards associations with mental health (Benach et al. 2015; Hult et al. 2023; Julià et al. 2017; Vives et al. 2011, 2013). Similar associations have been documented using related proxy indicators (Gevaert et al. 2021; Peckham et al. 2019). Among domestic cleaners, mental health complaints have been linked to precarious employment in the USA (Baron et al. 2022). Moreover, there are a few studies supporting a longitudinal relationship between multidimensional indicators of precarious employment and adverse mental health (Canivet et al. 2016; Eisenberg-Guyot et al. 2020).

Three main mechanisms linking precarious employment and mental health can be assumed. First, direct psychological effects associated with ‘the precarity of work’ can be assumed: i.e. experiences related to uncertainty and insecurity about one’s immediate and long-term occupational future (Blake et al. 2021). This includes perceived job insecurity and feelings of uncertainty, powerlessness and injustice about employment-related issues such as schedules, pay or organizational decisions (Bosmans et al. 2015; Vanroelen et al. 2021). *Therefore, we hypothesize that precarious employment—as well as its different constituting dimensions—is positively associated with adverse mental health among SVS workers.*

The second mechanism is of a more indirect nature and is termed ‘precarity at work’ by Blake et al. (2021). Precarious workers are more prone to adverse task-intrinsic job characteristics, like psychosocial demands, harsh physical working conditions or adverse social relations (Bosmans et al. 2016; Van Aerden et al. 2014). Moreover, also a higher exposure of precarious workers to workplace health and safety risks has been reported (Quinlan & Bohle 2004). This is particularly important for domestic cleaners, as employment relations characterized by insecurity, lack of voice and unequal power affect the workload and control over the work task. *We hypothesize that (a) precarious employment among SVS workers is positively associated with adverse task-intrinsic working conditions and that (b) part of the association between precarious employment and adverse mental health is mediated by these work task-intrinsic characteristics.*

Third, there is ‘precarity from work’ (Blake et al. 2021)—i.e. precarious employment tends to spill over to family’s socio-economic living situation in the form of material deprivation (Kretsos & Livanos 2016). At the level of the household, low work intensity of the partner, single parentship, or indebtedness in combination with a low paid job in the SVS system may lead to financial strain and material deprivation (Mousaid et al. 2017). Material deprivation and its related consequences have been linked to poor mental health (Kiely et al. 2015). SVS employment regularly serves as a kind of ‘adjustment factor’ in dual wage-earner households (Department_WSE 2020). Therefore, the material situation of SVS workers strongly depends of the wider household material situation (e.g. the presence of another breadwinner) (Mousaid et al. 2015). Therefore, household-level economic strain might be an important mediator of the association between precarious employment and mental health. *Based on this, it is our third hypothesis that (a) precarious employment among SVS workers is positively associated with the occurrence of financial strain at the household level and that (b) part of the association between precarious employment and adverse mental health is mediated by this household-level financial strain.*

## Methods

### Data and study population

Data were collected during 2019 in Belgium as part of a broader study devised to explore the quality of employment in the country. For this purpose, a survey including information on a wide range of employment-related aspects, demographics, socio-economic characteristics and health outcomes was performed. This survey was distributed in three languages (French, Dutch and English) among members and sympathizers of two divisions of the Belgian socialist trade union, ABVV. The result was a convenience sample, of which the details are discussed elsewhere (Vandevenne & Vanroelen 2020). Although the sample is not representative of the Belgian working population, it concerns a broad sample of 4,516 mainly working-class respondents, of which 2,707 were in paid employment. During the promotion campaign of the before-mentioned survey, the SVS group was highly mobilized, leading to a substantial sub-sample of SVS workers. This sub-sample was predominantly female (only 18 men). Therefore, we decided to concentrate our analyses on a cross-sectional sample of 1,115 female SVS workers. This sample is comparable to the SVS population in terms of age structure and gender composition (only 2.6% in the population). However, our sample contains more full-time SVS workers (25.2%), compared to the population (6.6%). The sample is further described in Table 1. For the regression analyses, we worked with a complete case dataset (*n* = 877), based on listwise deletion of all respondents with at least one missing value in the final model. Listwise deletion did not introduce major bias.

**Table 1:** Description of the sample and of the main variables included in the model (absolute numbers, bivariate means and ANOVA tests for categorical differences)

	*N*	Precarious employment	Quantitative demands	Physical demands	Low task variation	Low autonomy	Financial strain	Adverse mental health (WHO5)
		(*N* = 980)	(*N* = 1035)	(*N* = 1043)	(*N* = 988)	(*N* = 1001)	(*N* = 986)	(*N* = 1016)
		Mean	Mean	Mean	Mean	Mean	Mean	Mean
Age		***			**	***		**
Under 25	42	0.36	0.70	0.82	0.46	0.54	0.67	0.55
25–34	258	0.37	0.69	0.81	0.52	0.44	0.62	0.51
35–44	343	0.37	0.67	0.80	0.53	0.38	0.65	0.50
45–54	335	0.33	0.67	0.79	0.47	0.37	0.63	0.47
55 +	137	0.32	0.64	0.75	0.47	0.36	0.60	0.43
Household status					*		***	**
Single	102	0.34	0.68	0.80	0.52	0.40	0.72	0.50
Single with children	139	0.35	0.68	0.80	0.52	0.43	0.73	0.52
Couple	120	0.34	0.67	0.81	0.45	0.38	0.56	0.47
Couple with children	534	0.36	0.68	0.80	0.51	0.39	0.61	0.49
Other or missing	220	0.34	0.65	0.76	0.46	0.38	0.58	0.42
Educational attainment					**			
No or primary degree	145	0.37	0.67	0.80	0.44	0.41	0.63	0.49
Lower secondary or equal	341	0.35	0.68	0.80	0.49	0.38	0.62	0.48
Higher secondary	432	0.35	0.69	0.80	0.52	0.39	0.63	0.48
Tertiary	65	0.35	0.61	0.73	0.57	0.45	0.63	0.50
Other or missing	132	0.35	0.63	0.77	0.48	0.38	0.70	0.50
Working hours		***						
Part-time < 21 h	251	0.39	0.66	0.79	0.50	0.38	0.62	0.48
Part-time 21–32 h	582	0.35	0.67	0.79	0.51	0.40	0.63	0.49
Full-time (33 +)	280	0.32	0.68	0.81	0.47	0.38	0.64	0.47

Source EPRES-BE survey (own analyses); *p < 0.05, **p < 0.01, ***p < 0.001; all scales range from ‘0’ to ‘1’

The fieldwork underlying this study has been approved by the Ethical Committee of the Human Sciences of Vrije Universiteit Brussel (advice number ECHW_172.02). The dataset underlying the information in Tables 1, 2, 3, 4, annex 1 and annex 2 is available from the corresponding author (Christophe Vanroelen—christophe.vanroelen@vub.be) on reasonable request.

**Table 2:** Bivariate correlations between the precarious employment scale, the scales representing work task-intrinsic characteristics, perceived financial strain and adverse mental health

	Precarious employment	Quantitative demands	Physical demands	Low task variation	Low autonomy	Financial strain	Adverse mental health (WHO5)
Precarious employment	1.000	0.278***	0.190***	0.187***	0.409***	0.241***	0.274***
Temporariness		*0,024*	− *0,001*	*− 0,039*	*0,062*	*0,004*	*0,033*
Disempowerment		0,226***	0,140***	0,173***	0,379***	0,215***	0,265***
Vulnerability		0,377***	0,238***	0,165***	0,439***	0,273***	0,311***
Rights		0,690***	*0,047*	0,147***	0,114***	0,114***	0,120***
Enforceability of rights		0,096***	*0,056*	0,128***	0,194***	0,120***	0,139***
Working times		0,236***	0,126***	*0,049*	0,278***	0,174***	0,228***
Wages		*0,029*	*0,048*	0,095***	*0,044*	*0,062*	0,078**
Training		*0,037*	*0,033*	*0,020*	0,103***	*0,031*	*0,017*
Quantitative demands		1.000	0.541***	0.019	0.314***	0.280***	0.392***
Physical demands			1.000	-0.024	0.167***	0.170***	0.286***
Low task variation				1.000	0.215***	0.117***	0.204***
Low autonomy					1.000	0.231***	0.299***
Financial strain						1.000	0.433***
Low mental health (WHO5)							1.000

EPRES-BE survey (own analyses); *p < 0.05. **p < 0.01. ***p < 0.001

**Table 3:** Bivariate and fully adjusted associations with adverse mental health (WHO5) of precarious employment, the mediating variables and control variables; indirect and total effects (Bèta estimates and standard errors (S.E.)

	Bivariate	Mediation model EPRES-dimensions^a^	Mediation model EPRES-scale^b^
	Bèta (S.E.)	Bèta (S.E.)	Bèta (S.E.)
Direct effects			
Constant		− 0.277 (0.171)	− 0.313 (0.165)
Age			
Under 25	0.134 (0.045)**	0,036 (0,038)	0.051 (0.037)
25–34	0.081 (0.028)**	0.019 (0.048)	0.037 (0.047)
35–44	0.086 (0.027)**	0.029 (0.052)	0.044 (0.050)
45–54	0.046 (0.027)*	0.011 (0.049)	0.019 (0.049)
55 +	Ref	Ref	Ref
Household status			
Single	0.036 (0.033)	− 0,015 (0,042)	− 0.019 (0.041)
Single with children	0.061 (0.031)**	− 0.008 (0,039)	− 0.013 (0.039)
Couple	Ref	Ref	Ref
Couple with children	0.024 (0.025)	0,004 (0,045)	− 0.009 (0.045)
Precarious employment	0.545 (0.063)***		0.066 (0.032)*
Temporariness	0.095 (0.058)	0,035 (0,026)	
Disempowerment	0.209 (0.026)***	0,067 (0,032)*	
Vulnerability	0.310 (0.034)***	0,002 (0,041)	
Rights	0.130 (0.038)**	0,031 (0,033)	
Enforceability of rights	0.289 (0.051)***	0,020 (0,032)	
Working times	0.449 (0.066)***	0,002 (0,041)	
Wages	0.039 (0.022)	0,012 (0,030)	
Training	0.012 (0.017)	− 0,020 (0,028)	
Quantitative demands	0.401 (0.032)***	0,195 (0,040)***	0.207 (0.038)***
Physical demands	0.320 (0.036)***	0,098 (0,036)**	0.097 (0.035)**
Task variation (low)	0.180 (0.031)***	0,120 (0,030)***	0.121 (0.029)***
Autonomy (low)	0.283 (0.030)***	0,078 (0,035)*	0.093 (0.033)**
Financial strain	0.430 (0.030)***	0,299 (0,032)***	0.306 (0.031)***
Indirect effects of precarious employment			
Via quantitative demands			0.056***
Via physical demands			0.019*
Via task variation (low)			0.019**
Via autonomy (low)			0.038**
Via financial strain			0.067***
Total indirect effect			0.200***
Share of indirect effects in total effect EPRES-BE			75,1%

EPRES-BE survey (own analyses); *p < 0.05, **p < 0.01, ***p < 0.001

**Fig. 1:**
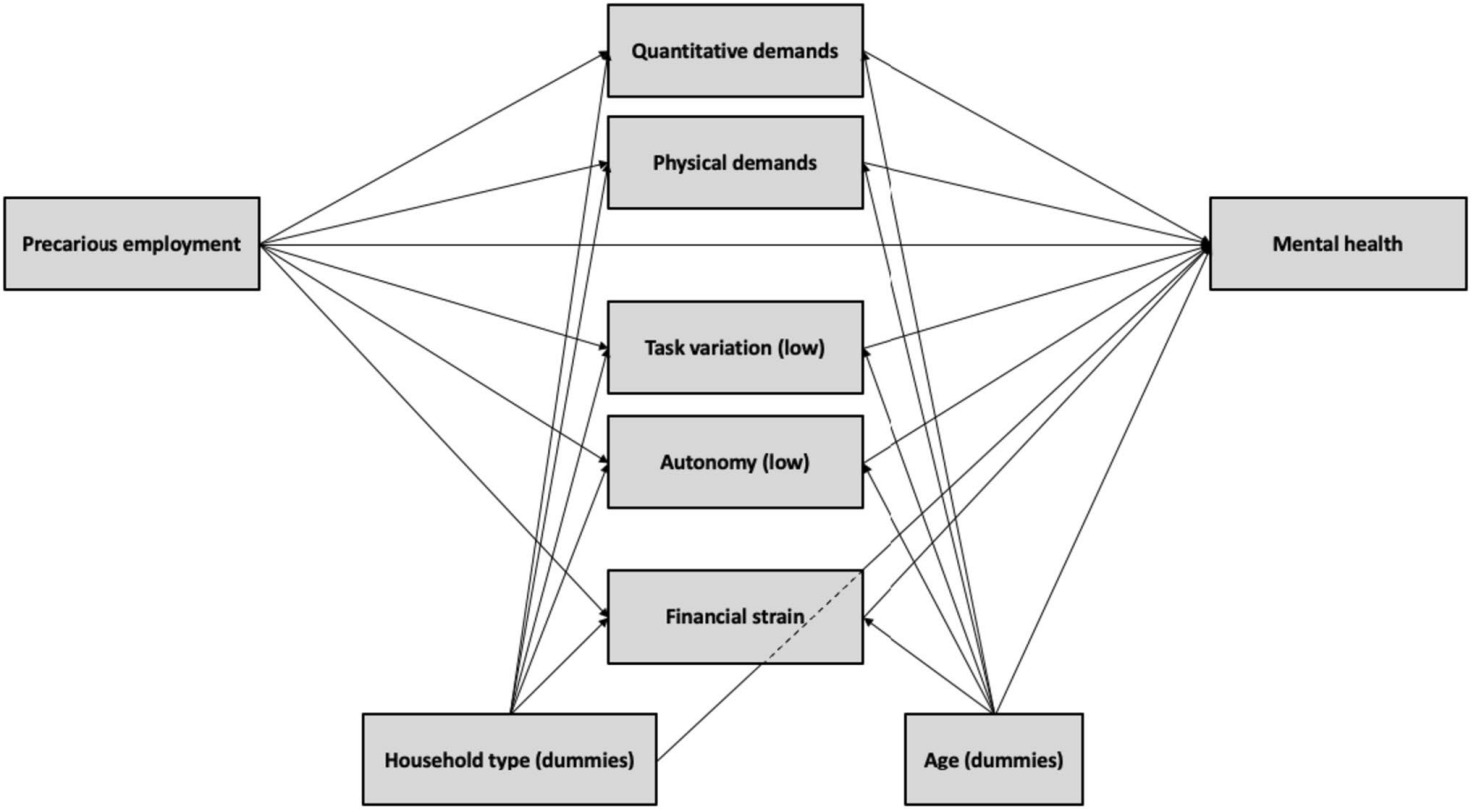
Stylized representation of the estimated mediation model

Bivariate models for all variables included separate in relation to mental health; Mediation model^a^: including the EPRES dimensions in a structural model with the same mediational pathways as specified in Fig. 1 model^a^, x—fit indices: Chi^2^ (*p* value): 0.000; CFI: 0.998; TLI: 0.885; RMSEA: 0.040; SRMR: 0.004; mediation model^b^: including the EPRES score in a structural model as specified in figure. x—fit indices: Chi^2^ (*p* value): 0.000; CFI: 0.998; TLI: 0.923; RMSEA: 0.038; SRMR: 0.006

**Table 4:** Bivariate and fully adjusted associations between age, household status and precarious employment and the mediator variables [Bèta estimates and standard errors (S.E.)]

	Quantitative demands		Physical demands		Low task variation		Low autonomy		Financial Strain	
	Bèta (S.E.)		Bèta (S.E.)		Bèta (S.E.)		Bèta (S.E.)		Bèta (S.E.)	
	Bivariate	Mediation model^a,b^	Bivariate	Mediation model^a,b^	Bivariate	Mediation model^a,b^	Bivariate	Mediation model^a,b^	Bivariate	Mediation model^a,b^
Age^(b)^										
Under 25	0.055 (0.033)	0.041 (0.034)	0.057 (0.033)	0.042 (0.034)	0.009 (0.040)	0.011 (0.041)	0.123 (0.035)***	0.102 (0.030)**	0.052 (0.038)	0.071 (0.038)
25–34	0.100 (0.053)	0.065 (0.055)	0.121 (0.052)*	0.094 (0.057)	0.123 (0.051)*	0.106 (0.054)*	0.124 (0.047)**	0.088 (0.048)	0.034 (0.053)	0.055 (0.055)
35–44	0.100 (0.056)	0.068 (0.058)	0.125 (0.054)*	0.105 (0.060)	0.154 (0.054)**	0.132 (0.057)*	0.028 (0.050)	-0.009 (0.053)	0.092 (0.056)	0.090 (0.057)
45–54	0.075 (0.056)	0.071 (0.057)	0.127 (0.054)*	0.125 (0.057)*	0.031 (0.054)	0.028 (0.054)	0.008 (0.049)	0.003 (0.048)	0.050 (0.056)	0.057 (0.055)
55 +	Ref	Ref	Ref	Ref	Ref	Ref	Ref	Ref	Ref	Ref
Household status^(b)^										
Single	0.025 (0.043)	0.032 (0.043)	− 0.011 (0.044)	− 0.002 (0.057)	0.095 (0.045)^*^	0.104 (0.045)^*^	0.013 (0.043)	0.035 (0.040)	0.204 (0.044)^***^	0.216 (0.043)^***^
Single with children	0.047 (0.045)	0.029 (0.044)	-0.013 (0.046)	-0.033 (0.048)	0.097 (0.045)^*^	0.081 (0.046)	0.058 (0.045)	0.064 (0.042)	0.262 (0.046)^***^	0.249 (0.045)^***^
Couple	Ref	Ref	Ref	Ref	Ref	Ref	Ref	Ref	Ref	Ref
Couple with children	0.042 (0.051)	0.010 (0.051)	− 0.022 (0.054)	− 0.054 (0.049)	0.133 (0.045)^*^	0.089 (0.046)	− 0.008 (0.051)	− 0.025 (0.048)	0.105 (0.055)	0.080 (0.046)
Precarious employment^(b)^	0.274 (0.032)***	0.271 (0.032)***	0.202 (0.032)***	0.200 (0.033)***	0.177 (0.034)***	0.159 (0.034)***	0.417 (0.028)***	0.409 (0.027)***	0.228 (0.034)***	0.220 (0.033)***
Temporariness^a^	*0.020 (0.017)*	*0.014 (0.034)*	*0.030 (0.052)*	*0.007 (0.030)*	− *0.066 (0.063)*	*− 0.093 (0.061)*	0.123 (0.061)*	*0,041 (0,031)*	*0.001 (0.065)*	− *0.011 (0.038)*
Disempowerment^a^	0.186 (0.026)***	0.118 (0.033)***	0.111 (0.023)***	0.073 (0.033)*	0.135 (0.029)***	0.096 (0.031)**	0.325 (0.026) ***	0,253 (0,030)***	0.154 (0.027)***	*0,093 (0,034)*
Vulnerability^a^	0.393 (0.033)***	0.298 (0.037)***	0.238 (0.031)***	0.173 (0.040)***	0.177 (0.040)***	0.141 (0.051)**	0.490 (0.034)***	0,269 (0,036)***	0.285 (0.035)***	0.180 (0.040)***
Rights^a^	*0.065 (0.035)*	*0.011 (0.033)*	*0.057 (0.033)*	*0.021 (0.034)*	0.164 (0.040)***	0.103 (0.040)*	0.126 (0.038)**	*0,029 (0,030)*	0.117 (0.038)**	*0,063 (0,035)*
Enforceability rights^a^	0.317 (0.049)***	*0.028 (0.037)*	0.237 (0.048)***	*0.076 (0.044)*	*0.075 (0.058)*	− *0.070 (0.065)*	0.477 (0.052)***	0,123 (0,034)***	0.263 (0.051)***	*0,042 (0,038)*
Working times^a^	0.536 (0.058)***	0.125 (0.032)***	0.262 (0.053)***	*0.035 (0.033)*	*0.089 (0.077)*	− *0.046 (0.083)*	0.554 (0.075)***	*0,048 (0,033)*	0.360 (0.065)***	0,069 (0,034)*
Wages^a^	*0.006 (0.022)*	− *0.034 (0.032)*	*0.016 (0.021)*	*0.004 (0.036)*	0.061 (0.024)*	0.050 (0.025)*	*0.029 (0.023)*	*− 0,003 (0,029)*	*0.029 (0.023)*	*0,042 (0,034)*
Training^a^	*0.020 (0.017)*	− *0.049 (0.032)*	*0.019 (0.016)*	− *0.007 (0.036)*	*0.021 (0.018)*	− *0.001 (0.018)*	0.062 (0.017) ***	*0.015 (0.015)*	*0.013 (0.017)*	− *0,042 (0,034)*

EPRES-BE survey (own analyses); *p < 0.05, **p < 0.01, ***p < 0.001

Mediation model^a^: including the EPRES dimensions in a structural model with the same mediational pathways as specified in Fig. 1 model^a^, x—fit indices: Chi^2^ (*p* value): 0.000; CFI: 0.998; TLI: 0.885; RMSEA: 0.040; SRMR: 0.004; Mediation model^b^: including the EPRES score in a structural model as specified in figure. x—fit indices: Chi^2^ (*p* value): 0.000; CFI: 0.998; TLI: 0.923; RMSEA: 0.038; SRMR: 0.006

### Variables

Our main dependent variable was mental health, which was measured using the five-item World Health Organization Well-Being Index (WHO5) (World Health Organization, 1998). The index consists of five positively worded statements (see table A.2) inquiring the interviewee’s well-being in the 2 weeks prior to the survey (α: 0.879) using a five-category Likert response scale ranging from ‘not at all’ to ‘always’. The responses were aggregated, and the resulting sum was standardized to a continuous scale with decimal values ranging from '0' to '1' (higher values indicate poorer mental well-being).

Precarious employment, our main independent variable, was measured using the EPRES-BE scale (see table A.1 for details on its construction). This scale is the Belgian adaptation of EPRES (Vives et al. 2010) and is composed of 38 items sorted into eight dimensions: (1) temporariness, (2) disempowerment, (3) vulnerability, (4) rights, (5) enforceability of rights, (6) working times, (7) wages and (8) training. Most main dimensions consist of sub-dimensions: e.g. the main dimension ‘working times’ is composed of the sub-dimensions ‘predictability of workings times’, ‘flexible working times’, ‘long working hours’ and ‘being stand-by’. The different survey items are quantified to a scale, with high values indicating a high degree of employment precariousness. All items constituting the EPRES-BE scale are coded as binary or ordinal variables ranging between ‘0’ and ‘1’. As a first step, the items are summed within their own sub-dimensions and standardized to a 0–1 range, then the sub-dimensions are summed within their main dimension and again standardized on a 0–1 range. Finally, the EPRES-BE scale is constructed by summing the main dimensions and once again standardizing them to a 0–1 range. While applying this stepwise approach, we checked the internal consistency between the items: items with a high Cronbach’s alpha were first summed together into a sub-dimension (see table A.1). There are some dimensions (working hours and rights) whose sub-dimensions did not have a Cronbach's alpha greater than 0.6. These sub-dimensions were still constructed because they were considered to belong together based on conceptual grounds (for example: weekend, evening and night work, each indicating flexible working times, but seldom occurring simultaneously in the same respondent). In “Results” associations are shown for the eight dimensions and for the overall EPRES-BE scale. In each case it concerns a continuous scale with values close to ‘0’ referring to the least precarious employment situations and values close to ‘1’ referring to the most precarious situations.

Several mediators are included in our analyses. First, physical demands are included as a summed scale based on three items (hard physical efforts, carrying heavy loads, work in uncomfortable positions—α: 0.712), with high scores pointing towards high physical demands. Psychosocial working conditions were measured, following the three main dimensions of Karasek’s demand–control model (Karasek 1979). Demands were conceived as ‘quantitative demands’ involving four items in a summed scale (having to work fast, having to work hard, not having too much workload, enough time to finish work—α: 0.782). High scores point towards high quantitative demands. Task variation is a summed scale consisting of five items (learning new things, need high professional competences, need creativity, having to do different things, possibility to learn new things—α: 0.756). High scores point towards low task variation. Autonomy is a summed scale consisting of three items (limited freedom to decide how to do things, having a lot to say, room for own decisions—α: 0.624). High scores point towards low autonomy. Each of the continuous scales was standardized to a ‘0’ to ‘1’ range. Detailed information on the scales can be found in table A.2).

Perceived material strain was measured using the proxy indicator: “when you think of your household’s total monthly income, can your household […] make ends meet?” (very easy, easy, fairly easy, with some difficulties, with difficulties, with great difficulties). The answers were standardized to an ordinal scale with a ‘0’–‘1’ range. High scores point towards high perceived material strain.

Control variables were age (under 25; 25–34; 35–44; 45–54; 55 +), household status (single without children, single with children, couple without children, couple with children), educational attainment (no diploma or low educational attainment, lower secondary, higher secondary, tertiary) and working hours (part-time < 21 h; part-time 21–32 h and full-time (> 33 h)/week). In the final models, only age and household status were maintained. The other controls were dropped due to a lack of effect on the dependent variable. The different variables are described in Tables 1 and 2.

### Statistical analyses

Descriptive statistics were performed (see Table 1). Additionally, mean EPRES-BE scores and mean scores for the scales representing work task-intrinsic characteristics, financial strain and mental health are calculated by categories of the control variables and submitted to an ANOVA test. The associations between the scales are represented by means of a correlation matrix (Table 2). The bivariate associations between precarious employment and mental health and between the work task-intrinsic characteristics and mental health are close to linear. The same holds for the associations between precarious employment on one hand and the work task-intrinsic characteristics and the indicator of perceived financial strain on the other hand.

Subsequently, a regression approach was adopted consisting of two steps. First, bivariate associations were estimated between the independent variable, precarious employment, all mediators and control variables and mental health. The ordinary least squares methods were used. Second, a mediation model (see Fig. 1) was estimated using a structural equation model with manifest variables. We started with a fully saturated model. Subsequently, non-significant covariations were relaxed step by step, while considering indicators of model fit—i.e. the Chi-squared test ( χ^2^), comparative fit index (CFI), Tucker–Lewis index (TLI), root mean square error of approximation (RMSEA) and standardized root mean square residual (SRMR) (Kline 2023). The mediation model was bootstrapped 10.000 times to estimate reliable standard errors. The bivariate and mediation results are presented in Tables 3 and 4 showing regression estimates of dummies for the categorical variables and for scales ranging from 0 and 1. Although bivariate regression estimates and estimates derived from an SEM procedure are strictly speaking not comparable, we opted to present them in the same tables given space concerns.

Descriptive analyses were performed using SPSS version 28, while the SEM model was estimated using Stata/MP 14.2.

## Results

### Descriptive analyses

In Table 1, the associations between the scales and the background variables are shown. Adverse mental health only showed to be statistically different across categories of age and household status: i.e. younger workers and workers without a partner have higher average adverse mental health scores. In table A.3., associations between the sub-dimensions of the EPRES-BE scale and background variables are shown.

In Table 2, the correlations between the EPRES-BE scale and its sub-dimensions, work task-intrinsic characteristics, financial strain and adverse mental health are shown. EPRES-BE shows significant positive associations with adverse mental health. Six of the eight sub-dimensions of EPRES-BE are positively related to adverse mental well-being. Notably, 'vulnerability' displays the most robust correlation (*r*^2^ = 0.311), while the relationships for 'temporariness' and 'training' do not reach statistical significance. In table A.1, correlations between mental health and the separate indicators constituting the EPRES-BE scale are shown. The EPRES-BE scale is positively correlated with high quantitative demands, high physical demands, low task variation, low autonomy and high financial strain. Also, significant positive correlations between most of the EPRES-BE sub-dimensions and task-intrinsic characteristics can be noted. Each of the work task-intrinsic characteristics and financial strain are correlated with adverse mental health.

### Bivariate associations

Bivariate models with adverse mental health (Table 3) show a strong, statistically significant association with precarious employment (ß 0.545–S.E. 0.063). Also, all EPRES-BE-dimensions demonstrate significant positive associations with adverse mental health, except for 'temporariness,' 'wages' and 'training.' The most substantial bivariate associations are observed for 'working times' (ß 0.449–S.E. 0.066) and ‘vulnerability’ (ß 0.310–S.E. 0.034). Each of the work task-intrinsic characteristics is positive and statistically significantly associated with adverse mental health. The same holds for high financial strain.

Associations with the mediation variables are reported in Table 4. Significant bivariate associations are seen between the age categories and physical demands (higher among those aged 25 and older, except aged 55 +), low task variation (higher among middle aged) and low autonomy (higher among younger workers). For household status, lower task variation is seen among singles, compared to couples, but most notable are the increased chances of encountering financial strain among singles and singles with children, compared to couples. Moreover, positive associations between EPRES-BE and each of the work task-intrinsic characteristics and financial strain are seen. The sub-dimensions of EPRES-BE show a mixed picture, with generally strong associations of ‘disempowerment’, ‘vulnerability’, ‘enforceability of rights’ and ‘working times’ with work task-intrinsic job characteristics and high financial strain.

### Mediation analysis

A stylized representation of the estimated mediation model is shown in Fig. 1. Given the significant mutual correlations, covariations between the work task-intrinsic indicators and financial strain are specified. Compared to a fully saturated model, only the covariations between low task variation and quantitative demands and between low task variation and perceived financial strain were relaxed. Based on the correlations retrieved, work task-intrinsic characteristics and financial strain have been specified as mediators between EPRES-BE and adverse mental health. The final measurement model has a good model fit [Chi^2^ (*p* value): 0.000; CFI: 0.998; TLI: 0.923; RMSEA: 0.038; SRMR: 0.006].

In the mediation models, the associations with adverse mental health of age and household status are not statistically significant. The association between EPRES-BE and adverse mental health is small, but statistically significant (ß 0.066–S.E. 0.032). Only the sub-dimension of disempowerment (ß 0.067–S.E. 0.032) shows a significant association with adverse mental health. Each of the mediators shows a statistically significant positive association with adverse mental health. For the mediation model with the overall EPRES-BE-scale significant associations with mental health are exist for (column 3 of Table 3): quantitative demands, ß 0.207–S.E. 0.038; physical demands, ß 0.097–S.E. 0.035; low task variation, ß 0.121–S.E. 0.029; low autonomy, ß 0.093–S.E. 0.033; and financial strain, ß 0.306–S.E. 0.031.

Associations with the mediation variables are reported in Table 4. Compared to the reference category of workers aged 55 years and older, workers aged 45–54 years report significantly higher physical demands (ß 0.125–S.E. 0.054); low task variation is significantly higher in the age groups 25–34 years (ß 0.106–S.E. 0.054) and 35–44 years (ß 0.132–S.E. 0.057); and low autonomy remains higher for those aged under 25 years (ß 0.102–S.E. 0.030). For household status, low task variation is higher among singles (ß 0.104–S.E. 0.045), compared to workers living as a couple. Singles (ß 0.216–S.E. 0.043) and singles with children (ß 0.249–S.E. 0.045) experience higher exposure to financial strain, compared to workers living as a couple. EPRES-BE shows moderately strong positive associations with each of the mediators: quantitative demands (ß 0.271–S.E. 0.032), physical demands (ß 0.200–S.E. 0.033), low task variation (ß 0.159–S.E. 0.034), low autonomy (ß 0.409–S.E. 0.027) and financial strain (ß 0.220–S.E. 0.033). For the EPRES-BE sub-dimensions the strongest associations with the work task-intrinsic characteristics are seen for ‘disempowerment’ and ‘vulnerability’. ‘Vulnerability’ and ‘working times’ are positively related to high financial strain.

### Direct versus indirect relations between precarious employment and mental health

The association of EPRES-BE with adverse mental health gets significantly reduced when included in the full mediation model (Table 3). About 50% of the total effect of the EPRES-BE score runs through the task-intrinsic mediators (quantitative demands, physical demands, low task variation and low autonomy) and 25% of the total effect of precarious employment runs through household-level perceived financial strain. Consequently, it can be concluded that the association of precarious employment with adverse mental health is largely mediated by the indicators included in our model. Financial strain is responsible for the strongest indirect effect (ß 0.067); the total indirect effect of precarious employment on adverse mental health is ß 0.200. Looking at the separate effects of the EPRES-BE sub-dimensions, largely the same picture emerges: only for ‘disempowerment’, a significant direct association with adverse mental well-being remains.

## Discussion

The results of this study offer support for the hypothesis that precarious employment—using the EPRES-BE-scale—is positively associated with adverse mental health. Looking at the sub-dimensions of EPRES-BE, ‘disempowerment’, ‘vulnerability’, ‘working times’ and ‘enforceability of rights’ showed the strongest bivariate effects on adverse mental health. In a sensitivity analysis (table A.1), we further associated the constituting items of EPRES-BE with mental health. Most main dimensions and items were positively associated with adverse mental health. Exceptions were temporariness and some rights (paid vacation and benefits). Our findings are well in line with those of other studies investigating the association between multidimensional precarious employment and mental health (Benach et al. 2015; Hult et al. 2023; Julià et al. 2017; Vives et al. 2011, 2013). Specifically for the group of domestic cleaners, in a US study several separate dimensions of employment precariousness were found to be related to stress and depression, i.e. ‘unfair wages’, ‘unsupportive or abusive relations with clients’, ‘language barriers with clients’ and ‘job-related financial insecurity’, while ‘working for an agency’ (as opposed to being self-employed) protected against adverse mental health (S. Baron et al. 2022).

In the mediation model, the remaining direct association between EPRES-BE and adverse mental health remained significant, but amounted only to 25% of the total effect. Of the sub-dimensions, only disempowerment showed a direct effect on adverse mental health. Likely ‘disempowerment’ comes closest to the root cause of precariousness: i.e. workers’ lack of bargaining power (Vanroelen et al. 2021). Furthermore, 'disempowerment' exhibits noticeable correlations with 'vulnerability,' 'working times,' and 'enforceability of rights,' indicating a degree of commonality. These findings might inspire researchers to adopt a more compact version of the EPRES-BE scale in the future. However, it is important to acknowledge that the current study was conducted within a highly specific and relatively homogeneous occupational group concerning certain employment conditions. It is noteworthy that the SVS population is primarily composed of individuals with permanent contracts, entitled to basic workers' rights such as a statutory minimum wage and paid vacations, and has full access to social security (RSZ 2022). Therefore, they likely present an atypical (precarious) worker population.

In line with our second hypothesis, we find evidence for the mediating role of work task-intrinsic characteristics. About 50% of the total effect of EPRES-BE runs through these factors (of which 21% via quantitative demands). In other words, domestic cleaners who find themselves in precarious employment have a high likelihood of being exposed to other adverse working conditions, jointly adding up to a considerable mental health penalty. Cross-sectional associations between precarious employment and adverse work task-intrinsic factors have been demonstrated earlier (Kvart et al. 2021; Peckham et al. 2019; Van Aerden et al. 2014). A recent paper using data from the European Working Condition Survey of 2015 reached similar conclusions as we did based on a mediation model including proxy indicators for employment precariousness and work task-intrinsic psychosocial risk factors (Méndez Rivero et al. 2021). Also, Baron et al. (2022) underline the importance of mediating mechanisms in explaining the relation between precarious employment and mental health among domestic workers. More specifically, the study points towards the mediating role played by exposure to adverse work-related irritant symptoms (mainly of the respiratory system and the skin), which could be seen as a proxy of harmful physical working conditions. Additional analyses (not shown) also underline the importance of these symptoms in our sample of domestic cleaners: 20% of our respondents report breathing difficulties and 60% report headaches or eye pains. Instead of health complaints, the mediators in our study were a series of work task-intrinsic characteristics with proven negative effects on mental health: harsh physical working conditions, high psychosocial demands, low autonomy and low task variation. The independent adverse effect on mental health of these work task-intrinsic characteristics—especially of quantitative demands—was confirmed. Quantitative demands have been highlighted as an important predictor of adverse (mental) health in cleaners previously (Hsieh et al. 2016).

The question remains whether the association between precarious employment and adverse work task-intrinsic factors reflects a ‘common cause’—i.e. a ‘bad job’ as it was called by Kalleberg (2016), or a causal chain in which precariousness results in adverse task-intrinsic working conditions. The design of the current study cannot resolve this issue. However, qualitative evidence is suggestive of the fact that task-intrinsic job characteristics are negatively affected by employment precariousness. Bosmans et al. (2015) describe how in mixed teams composed of temporary and permanent workers, the more dangerous and taxing tasks are distributed to the first. Also, Hill et al. (2019) reveal how precarious migration and employment situations of live-in caregivers in Canada tend to negatively affect psychosocial and physical hazards. Quinlan and Bohle (2004) have described how economic and reward pressures, workplace disorganization and regulatory failure endanger the occupational health and safety of precarious workers.

The third hypothesis, referring to the mediating role of household-level financial strain, can also be confirmed. Household-level financial strain showed a strong positive association with both precarious employment and adverse mental health. Therefore, 25%of the total association of EPRES-BE with adverse mental health ran through household-level perceived financial strain. A recent study using proxy indicators for precarious employment in the European Working Conditions Surveys of 2010 and 2015 also revealed a mediating role of household-level perceived financial strain (Pham 2022). The negative effect of precarious employment of one or two household members on the households’ financial situation is well established (Vanroelen et al. 2021). The median net monthly wage of the SVS workers in our sample is 1200 euros, which is well below the overall median wage levels in Belgium (3550 euros) (STATBEL 2022). While the hourly wages in the SVS are indeed low, the exceptionally low monthly earnings stem from part-time employment. These workers find themselves in a situation of ‘precarious unsustainable employment’ (Van Aerden et al. 2014). In other words, their employment generates insufficient economic revenue, making it unreliable for sustaining a decent living. When a precarious job is the only source of revenue from paid work in a household, ‘employment precariousness’ thus spills over into ‘social precariousness’. In our study, financial strain was particularly high among singles and singles with children. In these categories, (part-time) SVS employment is indeed likely the only source of revenue from paid work in the household, making financial strain almost inevitable. In contrast, Bosmans et al. (2017) have shown that other sources of income in a household (e.g. from a full-time and stable employed spouse) can have an important buffering effect on the harmful consequences of employment precariousness. This was likely the case for some of our respondents as well.

This study comes with certain limitations. First, the results are based on a relatively simple, cross-sectional study design based on a ‘convenience sample’. Because of its cross-sectional nature, formal causality statements cannot be made. This implies that the temporal order between exposure variable and mediators supposed for conducting a mediation analysis is based on theoretical assumptions and qualitative evidence (Bosmans, Hardonk, et al., 2016). The data used in the current study are part of a larger project in which the initial sample is longitudinally followed up. Future studies based on the EPRES-BE data will be able to formally test questions related to causality. Moreover, this study was based on a convenience sample established in collaboration with a trade union. Caution is warranted when using a convenience sample; however in specific circumstances, its use can be defended—i.e. when it comes to testing specific mechanisms in specific populations without claiming representativeness (Fabo & Kahanec 2018). This was the case in our study. Also, the high costs and complex contact procedures related to random sampling from official employment records and the desire to oversample precarious workers made us choose a convenience sample. Our trade union partner was a ‘trusted party’ making it easier to contact this hard-to-reach population. At the same time, 50% of all Belgian waged workers are unionized, with the socialist trade union being the second largest trade union, making it to a reliable sampling frame. The fact that the association between precarious employment and mental health found in our study is in line with results seen in other studies using EPRES is reassuring as well. Our questionnaire was available in French, Dutch or English—which are the main working languages for domestic cleaners in Belgium. For some potential respondents of migrant origin, however, filling out the questionnaire in one of these three languages might have posed an unsurmountable hurdle, leading to non-participation. A fourth set of limitations concerns the absence of some possible mediators. More specifically, it is known from previous research that domestic cleaners often consider themselves as working in social isolation—and that this can negatively affect their mental health (Bentein et al. 2017). Moreover, domestic cleaners, also SVS workers, tend to use ‘personalization strategies’ (i.e. becoming emotionally involved with clients as a strategy to limit exploitation) in their relations with their ‘client-employers’ (Safuta & Camargo 2019). A connection exists between precarious employment and the absence of support or unfavourable social relations (potentially resulting from personalization strategies) (Vanroelen et al. 2021). Therefore, it is likely that the quality of social relations mediates the association between precarious employment and mental health in our sample as well. However, as many domestic workers reported ‘missing’ or ‘not applicable’ when it came to the items on superior and co-worker support—probably they considered themselves as working alone—it was difficult to include these items in the analyses. Also controlling for prior mental health status was not possible, so we could not account for health selection effects.

A major strength of this study is that it tested the assumed mediating pathways linking precarious employment to adverse mental health. Therefore, it offers an important validation for the conceptual model linking employment precariousness to mental health that was proposed in the literature (Vanroelen et al. 2021). In addition, this is the first international paper reporting on the Belgian version of EPRES. EPRES-BE follows the tradition set out by Vives et al. (2010) for Spain and later replicated in several other countries. In doing so, it constitutes another important step in a movement towards reaching a common standard for measuring precarious employment in health and well-being research, which is recommended by central scholars in this field (Bodin et al. 2020). Nevertheless, the EPRES-BE instrument incorporates two additional dimensions—working times and training. The first dimension has been added to a revised version of the original EPRES-instrument upon the argument that flexible and irregular working times have been on the rise, are often imposed upon employees by their management and are limiting their control over work and social life (Padrosa et al. 2020). The second addition—access to training—is important in current knowledge-intensive twenty-first century labour market (Rubery et al. 2018). Therefore, EPRES-BE makes an important contribution to the further development of the EPRES approach. The results of our paper are also important for the specific population of domestic cleaners engaged in the Belgian SVS. The SVS offers formal employment, including mandatory worker rights and social security coverage, contractual security and convenient working hours, something that is hard to find in other low-skilled service occupations (Lens et al. 2022). Therefore, it is assumed to take away the sharp edges of employment precariousness for domestic workers. Still, SVS workers show relatively high average scores on the EPRES-BE scale (0.35, whereas 0.31 in the overall EPRES-BE sample). Moreover, a sub-sample of SVS workers shows quite some internal variation in terms of employment precariousness (S.D. of 0.122), even if the SVS employment scheme is devised to be rather homogeneous. High precariousness was furthermore strongly associated with adverse scores on intrinsic work task-related characteristics. This finding points at the importance for both working conditions and mental health of the employment characteristics underlying the notion of precariousness. A decomposition of the precariousness scale shows that SVS workers show the strongest associations with mental health for the dimensions ‘disempowerment’ and ‘vulnerability’ and for the sub-dimension ‘predictability of working hours’: e.g. how working times and schedules are determined, authoritarian treatment, access to information, whether wages and benefits are administrated well (see table A.1). So, it is clearly the quality of management of SVS agencies and—related to that—the relations with clients that make an important difference for employee well-being. The heterogeneity in employment relations in the SVS was already noticed in an earlier qualitative research (Mousaid et al. 2017). Moreover, the relationship between leadership styles, the quality of management and workers’ mental health is well established (Montano et al. 2017).

Therefore, our findings suggest that job quality improvements in the SVS can be reached by investing in more equal and participatory employment relations. Transformational leadership, high-quality relations-oriented and task-oriented leadership and high-quality interaction between managers and their employees are positively associated with mental health (Montano et al. 2017). ‘Holistic primary prevention interventions’ towards creating a more healthy psychosocial work environment in the SVS sector could concentrate on work organizational reforms (e.g. enhancing participation in decision-making, enriching job contents or more predictable scheduling), the quality of leadership (e.g. more rigour in correctly applying arrangements concerning rewards, social rights and scheduling; improving interpersonal communication; more regular site visits of clients’ premises) or engaging the clients (e.g. respectful communication, awareness training on unhealthy working conditions). Such recommendations have been made earlier (Mousaid et al. 2017), but seem still relevant.

A second important recommendation concerns the financial situation of SVS workers and their families. Policymakers should be aware of the inherent risk related to low-waged and ‘small’ jobs—especially in the case of the SVS, an employment scheme that is abundantly subsidized with public resources (Lens et al. 2022). To avoid the trap of in-work poverty, some policies with proven success are already in use in Belgium (e.g. supplementing part-time work with part-time unemployment benefits). However, given the strong public investment in the SVS, substantially higher hourly wages and collective working hour reduction could also be considered. Given the fact that, for many workers in the SVS, full-time work is simply not possible because of the harsh physical working conditions (Mousaid et al. 2017), decreasing a full-time working week (amounting to full wage and full social security entitlements) from 38 to 30 h would make a very positive contribution to the workability of SVS jobs. Also, policies helping those SVS workers who aspire to a professional future outside domestic cleaning by offering transferrable skills training and career counselling might help them achieve better paid employment.

In conclusion, this study among Belgian domestic cleaners engaged in the SVS revealed that only one sub-dimension of precarious employment—disempowerment—maintained a significant relation with mental health (ß 0.067) in a multivariable model simultaneously controlling for all other sub-dimensions of precarious employment, task-intrinsic working conditions and financial strain. The association of the overall EPRES-BE index with mental health was comparable to the effect of disempowerment (ß 0.066 vs ß 0.067). Most of the association between precarious employment and mental health runs indirectly through higher exposure to adverse work task-intrinsic job quality characteristics and perceived financial strain.

### Supplementary Information

Below is the link to the electronic supplementary material.

Supplementary file1 (DOCX 28 KB)

## Data Availability

The dataset underlying the information in Tables 1, 2, 3, 4, Annex 1 and Annex 2 is available from the corresponding author (Christophe Vanroelen – christophe.vanroelen@vub.be) on reasonable request.
