# Supplementary material for: Precarious employment and mental health in the Belgian service voucher system: the role of working conditions and perceived financial strain
Source: Int Arch Occup Environ Health. 2024 Mar 26;97(4):435–50. doi: 10.1007/s00420-024-02057-z (PMC10999388; doi:10.1007/s00420-024-02057-z)
Supplement: Supplementary file 1 — Supplementary file1 (DOCX 28 KB) [file 420_2024_2057_MOESM1_ESM.docx]

*Table Annex.1. Detailed description of the operationalization of the EPRES-BE instrument*

| **Dimension** | **Operationalisation** | **R^2^** ^($)^ | **Coding structure (0 = least prec. 1= most prec.)** |
| --- | --- | --- | --- |
| **Temporariness** | Do you have an open-ended contract with your employer? | 0.033 | 0. Open ended contract |
|  |  |  | 1. No open ended contract |
| **Disempowerment** |  | 0.265*** |  |
| ∑ (Formal empowerment, Informal empowerment) / 2 | Formal empowerment (α : 0.850) | 0.213*** | 0. Through… collective agreements |
|  | In which manner are the following employment conditions settled? |  | 0,33 negotiations between employee rep. and employer |
|  | *Hourly and monthly wages* | 0.191*** | 0,66. personal deliberation with my direct superior |
|  | *Social benefits and rights* | 0.206*** | 1. Imposed on me without any discussion + ‘I don’t know’ |
|  | Informal empowerment (α: 0.654) | 0.185*** | 0. I choose this myself + consultation with colleagues. |
|  | In which manner are the following employment conditions settled? |  | 0.5. In personal consultation with my direct superior. |
|  | *The work tasks of the day* | 0.186*** | 1. Imposed on me without consultation + I do not know. |
|  | *The weakly or monthly planning* | 0.131*** |  |
| **Vulnerability** |  | 0.311** |  |
|  | Authoritarianism (α: 0.860) | 0.304*** | 0. Totally disagree |
| ∑ (Autoritarian behaviour, lack of information), procedural justice) / 3 | *If I wanted better working conditions, I would be afraid to ask.* | 0.162*** | 0.25. Rather disagree. |
|  | *If I were to be treated unfairly, I would not dare to argue.* | 0.118*** | 0.5. Partly agree, partially disagree. + No opinion. |
|  | *I am treated in an authoritarian way.* | 0.195*** | 0.75. Rather agree |
|  | *I have to worry about getting fired if I don’t immediately do what told* | 0.240*** | 1. Totally agree. |
|  | *I would have to worry about being fired if I temporarily work less well.* | 0.247*** |  |
|  | *I would have to worry about getting fired if I take part in a strike.* | 0.206*** |  |
|  | *My bosses make me feel that I am easily replaceable.* | 0.341*** |  |
|  | *I am being treated aggressively* | 0.154*** |  |
|  | *I am being treated in an unfair or discriminatory manner.* | 0.215*** |  |
|  | Information |  | 0. Completely agree |
|  | *I’m well informed about health and safety risks associated with my job.* | 0.227*** | 0.25. Rather agree |
|  | Procedural justice |  | 0.5. Partly agree, partially disagree. + No opinion. |
|  | *The administration of my wages and is generally correct.* | 0.209*** | 0.75. Rather disagree. |
|  |  |  | 1. Completely disagree |
| **Rights** | I am entitled to … | 0.120*** | 0. Yes |
|  | *unemployment compensation in case of dismissal.* | 0.146*** | 1. No + I don’t know |
|  | *hospitalization insurance provided by my employer* | 0.053* |  |
|  | *1 benefit of: eco vouchers, meal vouchers, gift vouchers* | 0.001 |  |
|  | *paid vacation* | 0.046 |  |

| **Dimension** | **Operationalisation** | **R^2^** ^($)^ | **Coding structure (0 = least prec. 1= most prec.)** |
| --- | --- | --- | --- |
| **Enforceability of Rights** | Enforceability of six rights (α: 0.673) | 0.139 | 0. Completely agree + Rather agree |
|  | *I can actually… get my paid vacation without any problem.* | 0.148*** | 0.5. Partly agree, partially disagree |
|  | *get my paid sick leave without any problem.* | 0.087* | 1. Rather disagree + Completely disagree |
|  | *obtain thematic leave without any problem.* | -0.056* |  |
|  | *If I unexpectedly had to take a day off, that wouldn’t be a problem.* | 0.118 |  |
|  | *I am entitled to at least 1 of the following fringe benefits: eco vouchers,*  *meal vouchers, gift vouchers and these were paid / awarded to me*  *automatically and without any problems.* | 0.092** |  |
|  | *I am entitled to at least 1 of the following payments: bonus for working*  *in shifts, commuting allowance, bonus for hazardous work and these*  *were paid to me automatically and without any problems.* | 0.098** |  |
| **Working times** |  | 0.228*** |  |
| ∑ ((un)predictability + unsocial+ long overwork + stand-by) /4 | Predictability of working hours (Combined variable) | 0.210*** | 0.Schedule does not change regularly outside my control |
|  | *Does your work schedule change regularly beyond your control?* |  | 0.25. Change communicatedfew weeks in advance |
|  | *And how long before the change are you being notified?* |  | 0.5. Change communicated few days in advance ; no opinion |
|  |  |  | 0.75. Change communicated one day in advance |
|  |  |  | 1. Change communicated the same day |
|  | Unsocial and flexible hours (α: 0.523) | 0.099** | 0. Never. + I don't know + N / A. |
|  | *I work ... between 5 pm and 10 pm.* | -0.011 | 0.33. Sometimes |
|  | *at night" – i.e., between 8 pm and 5 am* | -0.022 | 0.66. Regularly. |
|  | *on a Saturday* | 0.004 | 1. Always. |
|  | *on a Sunday* | 0.035 |  |
|  | *on a holiday* | 0.004 |  |
|  | *Can you chose your start and finishing times (within certain limits)?* | 0.129*** | *0. Yes* |
|  |  |  | 1. No |
|  | Long hours/overwork (α: 0.046) |  |  |
|  | *I have to ... work overtime* | 0.150*** | 0. Never. + I don't know + N / A. |
|  |  |  | 0.33. Sometimes |
|  |  |  | 0.66. Regularly. |
|  |  |  | 1. Always. |
|  | *How many hours on average do you work per week in reality?* | 0.018 | 0. <40 h/week |
|  |  |  | 0,5. 41-48 h/week |
|  |  |  | 1. > 48 h/week |
|  | I have to be stand-by | 0.066* | 0. Never. + I don't know + N / A. |
|  |  |  | 0.33. Sometimes |
|  |  |  | 0.66. Regularly. |
|  |  |  | 1. Always. |

| **Dimension** | **Operationalisation** | **R^2^** ^($)^ | **Coding structure (0 = least prec. 1= most prec.)** | |
| --- | --- | --- | --- | --- |
| **Wages** | What amount best matches your own monthly net income from the main job? | 0.078* | 0. Twee highest quartiles | |
|  |  |  | 0,5. Second quartile. | |
|  |  |  | 1. Lowest quartile. | |
| **Training** | I have received training, paid or provided by my employer in the past 12 m. | 0.017 | 0. Yes | |
|  |  |  | 1. No. | |
| **Summed scale employment precariousness** | | 0.274*** |  |  |

*P-values R^2^: *p<0.05, **p<0.01, ***p<0.001; ^($)^Refers to the correlation with adverse mental well-being for each of the 38 items, sub-dimensions and main dimensions of EPRES-BE.*

*Table Annex.2. Detailed description of the operationalisation of the mental health and work-task intrinsic instruments*

| **Scale** | **Items** | **Cronbach's alpha** |
| --- | --- | --- |
| Mental health (WHO-5) | I felt happy and in high spirits | 0.879 |
|  | I felt calm and relaxed |  |
|  | I felt active and purposeful. |  |
|  | I felt refreshed and rested when I woke up |  |
|  | My daily life was filled with things that interested me |  |
| Physical demands | My job requires a lot of physical effort | 0.712 |
|  | My job often requires me to move or lift heavy loads |  |
|  | I often have to work in an uncomfortable position for long periods of time |  |
| Quantitative demands | My job requires me to work very fast | 0.782 |
|  | My job requires me to work very hard |  |
|  | I do not need to do an excessive amount of work |  |
|  | I have enough time to get my work done |  |
| Low task variation | My job requires me to learn new things | 0.756 |
|  | My job requires a high level of professional competence |  |
|  | My job requires me to be creative |  |
|  | I get to do many different things at my job |  |
|  | I have the opportunity to learn things at work |  |
| Low autonomy | I have little freedom to decide how I do my work | 0.624 |
|  | I have a lot of say in what happens at work |  |
|  | My job gives me room to make many decisions myself |  |

*Table A.3. Association between EPRES-subscales and background characteristics (absolute numbers, means and ANOVA-tests for categorical differences)*

|  |  | Precarious employment | Tempo-rariness | Disempowerment | Vulnerability | Rights | Enforcebility of rights | Working times | Wages | Training |
| --- | --- | --- | --- | --- | --- | --- | --- | --- | --- | --- |
|  | N | (N=980) | (N=1,115) | (N=1,115) | (N=1,115) | (N=1,115) | (N=1,112) | (N=1,045) | (N=983) | (N=1,114) |
| **Age** |  | *** | ** | *** | ** | *** | ** | ** | ** | ** |
| under 25 | 42 | 0,36 | 0,07 | 0,63 | 0,42 | 0,46 | 0,06 | 0,16 | 0,61 | 0,48 |
| 25-34 | 258 | 0,37 | 0,03 | 0,54 | 0,38 | 0,48 | 0,06 | 0,12 | 0,63 | 0,64 |
| 35-44 | 343 | 0,37 | 0,03 | 0,51 | 0,36 | 0,48 | 0,06 | 0,11 | 0,61 | 0,68 |
| 45-54 | 335 | 0,33 | 0,00 | 0,46 | 0,33 | 0,41 | 0,06 | 0,11 | 0,62 | 0,65 |
| 55+ | 137 | 0,32 | 0,01 | 0,39 | 0,28 | 0,38 | 0,05 | 0,10 | 0,65 | 0,67 |
| **Household status** |  |  | ** |  |  | ** |  | ** | *** |  |
| Single | 102 | 0,34 | 0,03 | 0,48 | 0,35 | 0,40 | 0,05 | 0,12 | 0,53 | 0,69 |
| Single with children | 139 | 0,35 | 0,01 | 0,54 | 0,36 | 0,46 | 0,06 | 0,12 | 0,54 | 0,68 |
| Couple | 120 | 0,34 | 0,03 | 0,51 | 0,34 | 0,40 | 0,06 | 0,13 | 0,59 | 0,59 |
| Couple with children | 534 | 0,36 | 0,02 | 0,48 | 0,35 | 0,46 | 0,06 | 0,11 | 0,67 | 0,64 |
| Other or Missing | 220 | 0,34 | 0,00 | 0,48 | 0,33 | 0,44 | 0,05 | 0,12 | 0,62 | 0,67 |
| **Educational attainment** |  |  |  |  |  |  |  |  |  |  |
| No or primary degree | 145 | 0,37 | 0,03 | 0,53 | 0,36 | 0,45 | 0,06 | 0,11 | 0,61 | 0,71 |
| Lower secondary or equal | 341 | 0,35 | 0,01 | 0,49 | 0,35 | 0,43 | 0,06 | 0,12 | 0,63 | 0,65 |
| Higher secondary | 432 | 0,35 | 0,03 | 0,47 | 0,34 | 0,45 | 0,06 | 0,11 | 0,64 | 0,62 |
| Tertiary | 65 | 0,35 | 0,15 | 0,45 | 0,34 | 0,45 | 0,05 | 0,12 | 0,59 | 0,72 |
| Other or Missing | 132 | 0,35 | 0,00 | 0,54 | 0,35 | 0,44 | 0,06 | 0,12 | 0,58 | 0,65 |
| **Working hours** |  | *** |  |  |  | ** |  |  | *** | ** |
| Part-time <21h | 251 | 0,39 | 0,04 | 0,49 | 0,34 | 0,47 | 0,05 | 0,11 | 0,91 | 0,64 |
| Part-time 21-32h | 582 | 0,35 | 0,02 | 0,50 | 0,35 | 0,45 | 0,06 | 0,11 | 0,66 | 0,62 |
| Full-time (33+) | 280 | 0,32 | 0,11 | 0,49 | 0,35 | 0,42 | 0,06 | 0,12 | 0,30 | 0,73 |
| *Source: EPRES-be survey (own analyses); *p<0.05, **p<0.01, ***p<0.001; all scales range from ‘0’ to ‘1’* | | | | | | | | | | |
